# Modified Free Gingival Autograft: A Case Report

**DOI:** 10.7759/cureus.45920

**Published:** 2023-09-25

**Authors:** Manpreet Singh, Renuka Renuka, Puneet Kamal Nagi, Azhagu Mani Malar K. I., Yerubandi Chandini Lakshmi

**Affiliations:** 1 Periodontics, Punjab Government Dental College and Hospital, Amritsar, IND; 2 Prosthodontics, Himachal Institute of Dental Sciences, Paonta Sahib, IND; 3 Oral Pathology and Microbiology, Vivekanandha Dental College for Women, Elaiyampalayam, IND; 4 Dentistry, Panineeya Institute of Dental Sciences & Research Centre, Hyderabad, IND

**Keywords:** surgical approach, recession, marginal, gingiva, free gingival graft

## Abstract

The purpose of this case study was to introduce an innovative method utilizing a modified free gingival graft technique, with the goal of accomplishing vertical and horizontal augmentation of soft tissue in the mandibular anterior region. A 35-year-old female patient underwent the modified free gingival graft technique in the anterior mandibular area. Remarkably, after a span of nine months, an increase was noted in both horizontal and vertical dimensions of the gingival tissue. This transformation was met with satisfaction from the patient. Following the successful graft procedure, a vertical gain of 3 mm was observed in the keratinized gingiva.

## Introduction

A captivating smile serves as the most exquisite adornment to one's countenance and stands as a primal mode of human interaction. The symphony of this smile is notably orchestrated by the contours, arrangement, and shade of the teeth [1]. Irrespective of age, individuals are affected by the allure of their smile and its broader aesthetic impact [2].

A robust mucogingival complex, wherein the mucogingival tissues preserve their inherent biomorphic integrity and secure a sustained connection to both the teeth and underlying soft tissue, remains an indispensable facet [3]. When a mucogingival issue arises, it typically manifests in two primary forms: (i) proximal disruption resulting in the formation of pockets [4] and (ii) an exposed disruption leading to the appearance of both gingival clefts and gingival recession [5].

Gingival recession is distinguished by the marginal gingiva moving downward in relation to the cementoenamel junction which can result in aesthetic concerns, heightened tooth sensitivity, susceptibility to root decay, and increased blood flow to the dental pulp [6]. Although the aetiology of gingival recessions remains unclear, several predisposing factors have been suggested. A high proportion of individuals with gingival recessions in populations with high standards of oral hygiene implies that mechanical and anatomic factors likely play a role. An “improper” toothbrushing method has been proposed as the most important mechanical factor contributing to the development of gingival recessions. Among anatomical variables, a thin gingival biotype and a reduced thickness of the alveolar bone due to abnormal tooth position in the arch, individual tooth shape, the presence of dehiscence/fenestration, or an aberrant path of eruption seem particularly relevant [7].

The indications for the root coverage procedures when gingival recession is seen include demands from the patient to enhance esthetics and reduce sensitivity due to root exposure. Furthermore, it enhances the quantity and dimensions of keratinized tissue, aiding in infection control [8,9]. Notably, patients with a history of prior orthodontic treatment often present with this issue in mandibular incisors [10]. This might be attributed to factors such as marginal frenulum attachment, heightened muscle pull, and a shallow vestibule.

Numerous surgical approaches exist for addressing gingival recession, each displaying varying degrees of success and predictability [11]. These encompass methodologies like free grafts, which include procedures such as free gingival grafts (FGGs) and subepithelial connective tissue grafts. Additionally, pedicled grafts like lateral and coronal grafts are utilized. The concept of FGGs was initially introduced by Bjorn in 1963 [12] and later labeled as FGG by Nabers [13]. As time progressed, FGGs evolved beyond merely covering exposed tooth roots to being employed to augment the width and thickness of attached gum tissue. The advantages associated with FGGs include their notable success rate and relatively uncomplicated implementation. However, it's important to acknowledge that conventional FGGs do have certain limitations, including the potential for aesthetic discrepancies and a potentially bulky appearance [14].

## Case presentation

**Figure 1:**
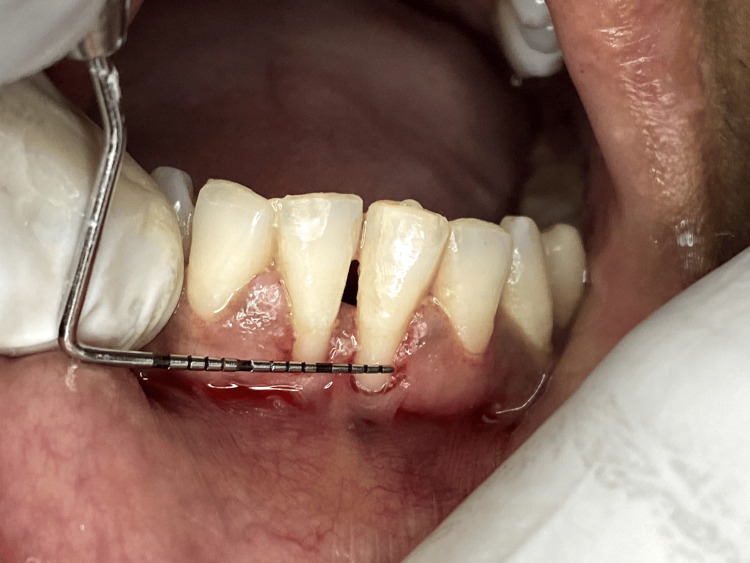
Miller class III defect with recession width of about 3 mm before procedure

**Figure 2:**
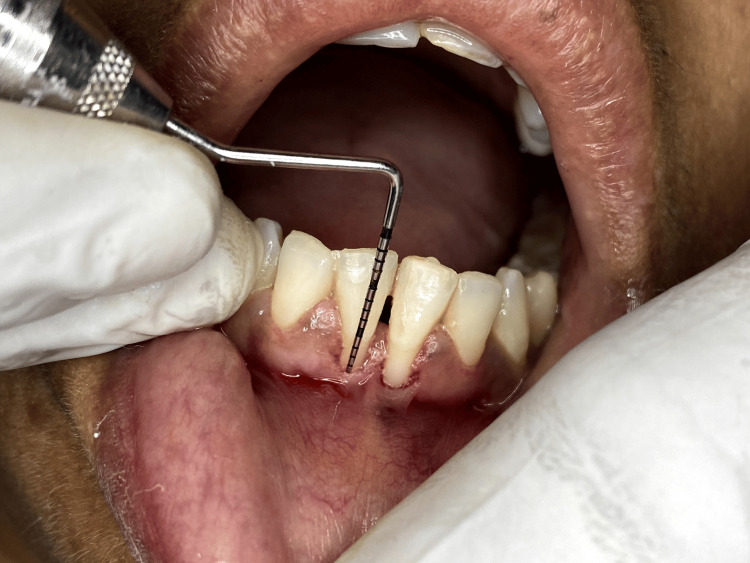
Miller class III defect with recession depth of 3 mm before procedure

A 35-year-old female patient presented with a chief complaint of gingival recession in the lower incisor region. She had no relevant medical or dental history. She also had no smoking or any drug history. She had an improper brushing habit. On clinical examination, the recession was manifested as a Miller Class III defect located within the mandibular incisor region (Figures 1, 2). The recession exhibited dimensions of 3 mm in width and 3 mm in depth with a thin gingival biotype. Just prior to the commencement of the procedure, the patient was asked to rinse the mouth for two minutes with a solution containing 0.12% chlorhexidine digluconate. After ensuring a sterile environment, a local anesthetic containing 2% lidocaine with 1:100,000 epinephrine was administered. Following that, a releasing incision was carried out, strategically located between the mucogingival junction and the marginal tissue. Subsequently, a partial-thickness flap was meticulously elevated, with the aim of maintaining proximity to the periosteum to ensure optimal bed preparation.

**Figure 3:**
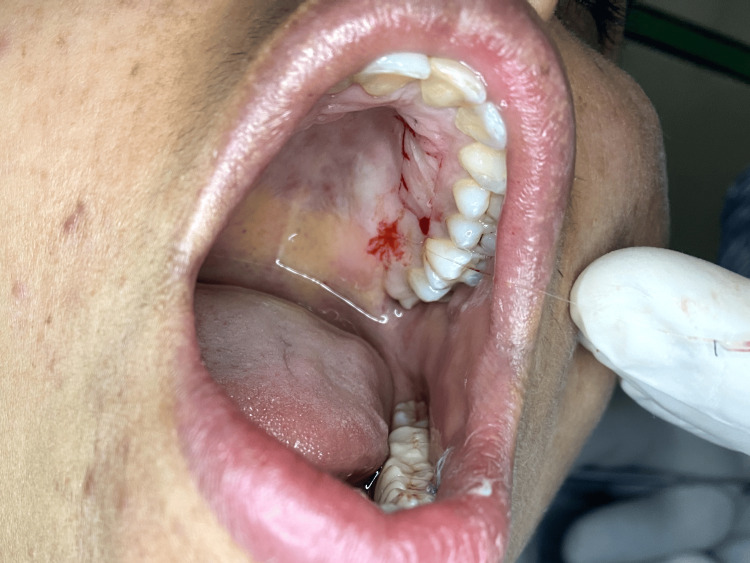
Donor site, which was at the left side of the anterior hard palate from the canine to second premolar of the maxilla.

**Figure 4:**
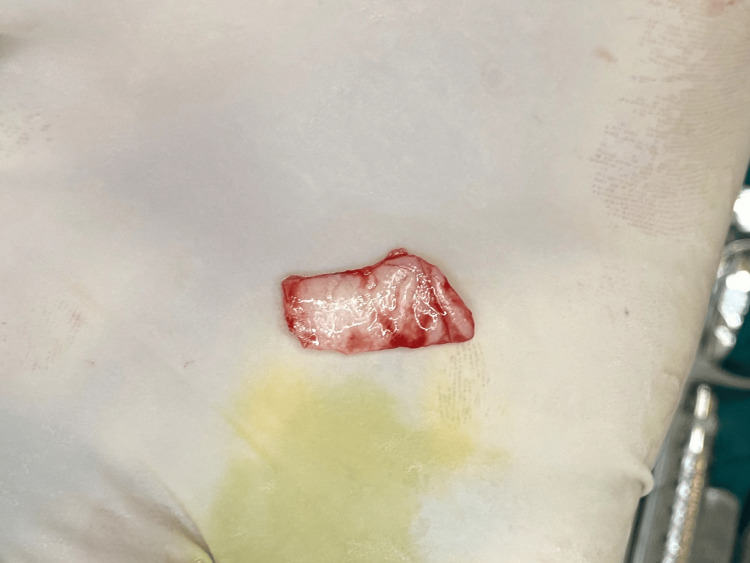
Harvested free gingival graft

**Figure 5:**
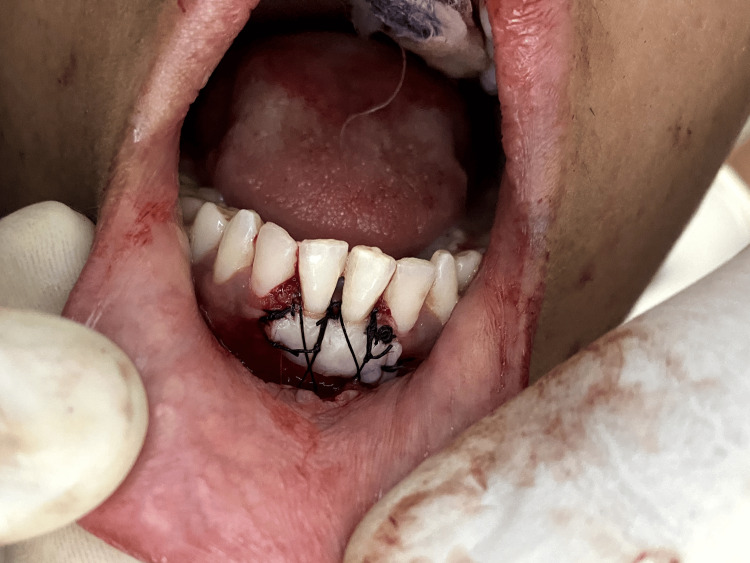
Recipient site after procedure

From the donor site, which is the left side of the anterior hard palate in the region extending from the canine to the second premolar of the maxilla, an FGG of thickness 1.5 mm was meticulously procured according to the size of the recipient site, which was measured using a foil template (Figures 3, 4). Using absorbable 6-0 Vicryl suture made from the copolymer of glycolic acid and lactic acid, the partially elevated flap was carefully placed in an apical direction and attached to the periosteum with simple interrupted sutures. Subsequently, the graft was placed with meticulous care onto the sturdy and resilient periosteal bed, orienting the connective tissue side towards the periosteum. This positioning was executed to guarantee that approximately 3.5 mm of periosteum remained exposed. Sutures with a thickness of 5-0 were employed to secure the graft (Figure 5). The suturing technique of Holbrook and Oschenbein was used.

Following the procedure, the patient adhered to a standard postoperative regimen, which included taking amoxicillin 500 mg three times a day for five days, aceclofenac 100 mg twice a day for five days, and using a 0.12% chlorhexidine digluconate mouthwash three times daily for four weeks. During the initial four weeks of the postoperative period, the patient was advised to refrain from chewing or brushing the surgical area.

**Figure 6:**
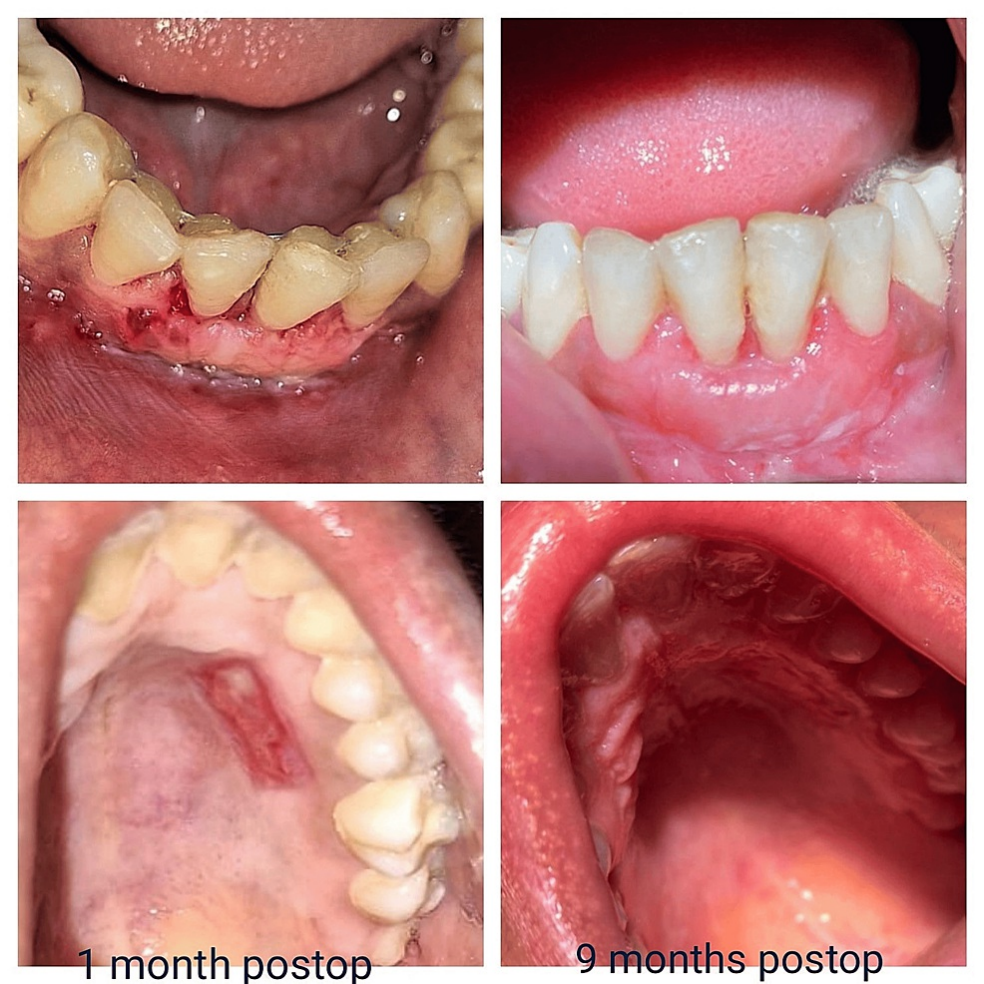
Recipient and donor site at the one-month follow-up and nine-month follow-up

The sutures were removed after 10 days. A comprehensive follow-up of both the recipient and donor site was done after one month. After nine months, another follow-up was conducted to evaluate the prognosis and outcomes of the procedure (Figure 6), in which a reduction in recession from 3 mm to 0.2 mm in height and 0 mm in width was noted.

## Discussion

The technique of FGG has consistently stood as a dependable and firmly established method for augmenting keratinized gingival tissue, serving to mitigate potential hard and soft tissue challenges following dental implant restoration [5]. Nonetheless, this technique involves dual surgical sites, posing potential discomfort and pain to patients. Moreover, variations in color and texture compared to the surrounding mucosa, along with a degree of graft shrinkage, can lead to suboptimal aesthetic outcomes [6,7]. This approach embraces a dual-component strategy that can be seen as a modification to the FGG procedure. In this procedure, one graft segment undergoes a process of de-epithelialization and is situated beneath the mucosal layer that encases the bone crest, facilitating vertical augmentation. Concurrently, another segment of the graft is positioned on the vestibular flap, promoting the lateral expansion of keratinized mucosa. [14].

This innovative method provides an advantage over traditional FGG by concurrently addressing both vertical and horizontal tissue augmentation. The placement of a graft portion on the bone crest contributes to vertical and horizontal tissue gain, a feature not commonly seen in classical approaches.

There were several recent studies that discussed the management of recession in the mandibular anterior teeth region. A study by Sculean et al. used enamel matrix derivative and connective tissue graft in the recession area by modified coronally placed tunnel technique and found an increase in keratinized tissue height of 0.5 mm and 96.3% root coverage [15]. A study by Sculean and Allen used a laterally placed tunnel technique for managing mandibular anterior recession and found an increase in keratinized tissue width of 2.8 mm and 96% root coverage [16]. A study by Carranza et al. [17] and Stefanini et al. [18] also found improved root coverage of 98.3%. A study by Carcuac et al. used a modified FGG in the mandibular anterior tooth region and found an increased keratinized tissue height of 4.2 mm [19], which was considerably greater than the earlier studies.

In the present case, we observed a higher prevalence of Miller class III recessions, which were attributed to improper toothbrushing techniques along with Miller class II. Conversely, Miller class I recessions were identified as a result of plaque accumulation. It is important to emphasize that the arrangement of gingival recessions has been linked to varying causative elements. It's worth noting that gingival recessions on the mandibular incisors are primarily associated with insufficient oral hygiene practices [20], whereas those affecting premolars may arise from aggressive brushing techniques [21].

An increased incidence of gingival recession was noted in males (60.5%) in contrast to females (39.5%) [20], confirming the alignment with prior research findings. Regions with insufficient keratinized mucosa have been demonstrated to exhibit greater vulnerability to gingival recession due to the limited presence of connective tissue in those areas. This deficiency gives rise to localized inflammatory reactions, triggered by various mechanisms that impact the entire tissue region, ultimately resulting in the development of gingival recession [20]. This phenomenon could potentially play a significant role in the occurrence of gingival recession, particularly in the anterior mandibular teeth.

The idea of gingival unit grafting introduces an innovative method that integrates both the marginal and papillary sections of the gingiva within the FGG procedure, departing from the conventional submarginal approach. The logic behind this concept is grounded in the heightened vascularity that is evident in the marginal and papillary gingival segments. This vascular richness can be attributed to the presence of a complex network of interconnected loops, hairpin-like structures, anastomoses, and a densely packed vascular plexus. The inclusion of this highly vascularized gingival segment in the graft yields enhanced integration with the recipient bed, accompanied by superior aesthetic coverage and a harmonious tissue blend. This application resulted in an exceptional tissue blend and color match, surpassing that attainable through conventional FGG techniques. Notably, the donor site exhibited no postoperative recession.

In cases where a partly epithelialized-subepithelial connective tissue graft is employed, the grafting procedure preserves islands of gingival epithelium that correspond to the dimensions of the recession. These epithelial islands safeguard the underlying connective tissue, potentially due to the epithelium's resilience in the oral environment. This approach offers benefits like maintaining the mucogingival junction position and preventing vestibule flattening. Despite its efficacy, the technique presents challenges, as retaining the epithelial islands results in increased healing time via secondary intention. Moreover, the meticulous nature of the procedure demands precise adherence of the epithelial islands to the recession defect's size.

The greater thickness of keratinized mucosa corresponds with enhanced oral hygiene, leading to diminished plaque buildup, reduced inflammation, decreased bleeding, and a lower risk of gingival recession [19]. Additionally, this thickness supports effective oral hygiene maintenance by patients [20].

The successful implementation of these modified FGG techniques hinges on meticulous case selection and precise tissue management. While promising, broader studies with larger participant cohorts are necessary to solidify the evidence supporting the effectiveness and applicability of these innovations.

The success of implementing these alterations to FGG relies on precise patient selection and meticulous handling of tissue. Various other factors include careful anatomical assessment of the amount of keratinized tissue in relation to the gingival defect, width of the defect, health of gingival tissue, modifying factors like diabetes and smoking, stabilization of graft and tension-free flap, thickness, and width of graft and frenal attachment level. Conducting additional studies with a more substantial participant pool would offer more definitive proof regarding the efficacy and suitability of these methods.

## Conclusions

This report highlights the effectiveness of the modified FGG technique, demonstrating its capacity to produce positive results by enhancing both vertical and horizontal dimensions of soft tissue. The results emphasize the successful retention of keratinized tissue, providing further evidence of the technique's efficacy. The unique approach of including both marginal and papillary gingival segments in the grafting procedure showcases encouraging results, particularly in terms of improved tissue integration, aesthetic improvements, and overall patient satisfaction. However, it's important to acknowledge the challenges associated with this technique, particularly in terms of increased healing time at the donor site. As with any innovative approach, proper patient selection and meticulous execution are vital for successful outcomes.
